# Supplementary material for: Energy drinks in Tamale: Understanding youth perceptions, consumption patterns, and related factors
Source: PLoS One. 2024 Apr 26;19(4):e0289391. doi: 10.1371/journal.pone.0289391 (PMC11051681; doi:10.1371/journal.pone.0289391)
Supplement: Attachment — Submitted filename: Response to Reviewers.docx [file pone.0289391.s001.docx]

**Title: Energy drinks in Tamale: understanding youth perceptions, consumption patterns and related factors**

**Manuscript Ref: PONE-D-23-21713**

We take this opportunity to thank the editor and reviewers of our paper for their kind collaboration to the improvement of this manuscript. We have taken into account all the concerns raised and we have made the suggested modifications. We have implemented numerous improvements to the paper based on these suggestions. Below, we justify our replies to the suggestions made by the respected editor and reviewers of the paper.

**EDITOR**

**Dear authors, we apologise for the delays in getting you feedback on your paper. We have now secured sufficient peer-review comments on your paper and are happy for you to revise based on their recommendations and submit for further evaluation. This paper is timely given the recent discussions from health and nutrition professionals in Ghana around soft and energy drinks consumption particularly among the youth. However, it is clear from the two reviewers and from my own assessment as Editor that, your paper needs substantial proofreading to tighten up the arguments and correct language errors. Also make sure you back all claims with sufficient evidence.**

Thank you for providing us with the feedback from both the reviewers and your own assessment. We appreciate the time and effort invested in reviewing our manuscript. We understand the importance of ensuring the quality and clarity of our work and are committed to addressing the highlighted concerns. Here is our response to the comments.

a. The paper has undergone substantial proofreading to tighten up arguments and correct language errors.
b. All claims are now backed by sufficient evidence, ensuring a more robust presentation.
1. **In addition to comments from the reviewers, consider reducing sections of the discussion and introduce a new section in the discussion on policy measures/recommendations for EDs in Ghana. After reading your findings, a good question is, what next? And so what so we do, in terms of policy or action? Can you link this discussion to the recently released Ghana Food Based Dietary Guidelines? What should be done around schools? What (healthy) alternatives are there and why do you think those will be chosen over energy drinks?**

We have reduced sections of the discussion and introduced a new segment focusing on policy measures and recommendations for energy drinks (EDs) in Ghana. This addition addresses the "what next" aspect and aligns the discussion with the recently released Ghana Food Based Dietary Guidelines. We have explored potential policy implications and actions, particularly in the context of schools, and discussed healthy alternatives to energy drinks. The suggestion titled ***Recommendations for ED Sale and Consumption*** is added in the discussion section (***see*** ***pgs. 20&21, line nos. 532-553***).

*In 2023, Ghana passed a tax bill on sugar sweetened-beverages into law. The law categorizes EDs as sugar sweetened drinks. The policy action is expected to increase individual savings and promote health [83]. Taxation is an effective strategy for reducing the consumption of sugar sweetened-beverages. While higher taxes have been more effective (above 10%), such initiatives have no significant impact in high-income countries compared to LMICs [84]. The extant tax regulations exhibit commendable strides in mitigating the consumption of EDs; however, there exists untapped potential for further reduction. It is imperative to reconsider the categorization of EDs given they differ from conventional sugar-sweetened beverages, as they have cardiovascular effects substantiated by several of compelling case studies [11,85–89]. This reclassification stands to facilitate a spectrum of viable interventions, encompassing but not confined to the following: stringent restrictions on the pervasive retail distribution of EDs; the implementation of rigorous age-related restrictions, and prohibition in schools governing the purchase and consumption of EDs; enhanced oversight of marketing, advertising, and product labelling; and the unequivocal disclosure of potential short- and long-term adverse effects associated with the consumption of EDs [6].*

*In alignment with the national food-based dietary guidelines, which stipulate that individual sugar consumption should not surpass 50 grams per day, there is a pressing need for heightened advocacy efforts aimed at fostering decreased sugar intake and curbing the consumption of sugar-sweetened beverages [90]. As EDs are prohibited in school environments, natural fruit drinks should be encouraged. This can be incorporated into the national school feeding program initiative. Additionally, it is essential to provide backing and incentives to beverage manufacturers to invest in the production of healthy, nutrient-rich beverages characterized by low sugar content.*

2. **What are the limitations of your study? Include this in the discussion. Reviewer 2 highlights some of these in different comments.**

Limitations of the study have been included in the discussion (***see pg. 21, line nos. 555-561*** ). We have elaborated on the potential shortcomings and challenges faced during the research process, providing a more comprehensive understanding of the study's scope and applicability.

*The adoption of a cross-sectional design restricts the establishment of causal relationships and temporal changes in ED consumption patterns and perceptions. The use of a convenience sample raises concerns about representativeness, potentially limiting the generalizability of findings. Self-report data may introduce recall bias and may not fully reflect actual behaviours and beliefs accurately. The choice of the study area based on personal familiarity introduces potential bias in the selection process. Face-to-face interviews might also introduce interviewer bias and compromise participant anonymity.*

**REVIEWER 1**

**This is indeed a very good and interesting Public health topic worthy of discussion. There are some suggestions which when added, can boost the strength of this research.**

Thank you for your thoughtful and constructive feedback on our research manuscript. We appreciate your positive remarks on the significance of the public health topic. We have carefully considered your suggestions and made the following revisions.

1. **What previous works have been done in Ghana regarding investigation of energy drink consumption?**

We have included a section summarizing previous works on energy drink consumption in Ghana in the introduction section (***see pg. 3, line nos. 84-86***)., identifying gaps, and highlighting the uniqueness of our study.

*This study builds upon existing research on ED consumption in Ghana, expanding beyond previous investigations that predominantly focused on specific cohorts like students, student-athletes, and drivers [19–24].*

2. **What gaps were identified?**

We have included some information in the introduction section on the identified gap (***see pg. 3, line nos. 86&88***). and highlighting the uniqueness of our study.

*However, they have left a gap in our understanding of ED consumption among the general youth population.*

3. **How unique is this work or how different is this work from the previous works?**

We have included some information in the introduction section on the identified gap (***see pg. 3, line nos. 87&88***). and highlighting the uniqueness of our study.

*This study addresses this gap by examining ED consumption among a representative sample of Ghanaian youth.*

4. **A map of Ghana showing Tamale and coordinates must be provided.**

A map of Tamale with a label showing the coordinates has been incorporated for better geographic context. We opted to use only the map of Tamale instead of the whole map of Ghana with the map of Tamale highlighted because in that view the Tamale area is not visible enough.

*Fig 1. Map of Tamale (Coordinates: 9.404601, -0.842389).*

5. **Last paragraph of the intro must be reconstructed for clarity
   “This study will make…..**

The last paragraph of the introduction has been revised for clarity, ensuring a more succinct expression of our study's aims and contributions (***see introduction, pg. 4, line nos. 91-93***).

*This study enhances our understanding of ED consumption habits among the youth in the Tamale Metropolis. The findings can raise awareness, prompt further research in this domain, and influence policy and public health initiatives.*

6. **Discussion seems summary of the whole report. Authors need to discuss key findings with sufficient literature**

We have expanded the discussion section to provide more in-depth analysis and interpretation of key findings, incorporating relevant literature to support our arguments.

***(See discussion section, pg. 15, lines 322-329)***

*This study found that the popularity of EDs is highly driven by advertising media, especially television. In support of this assertion, Stacey et al. [31] substantiated a correlation between the consumption of EDs and the exposure to promotional advertisements of ED brands. These advertisements were found to typically target a youthful male audience using sports and entertainment-centred themes [32], and leveraging associations with influential celebrities. Gendered advertising reflects the leveraging of higher involvement of males in sports. This is in line with an American study that strongly links ED consumption to traditional masculinity and risk-taking tendencies [33].*

(***See discussion section, pg. 16, line nos. 341-343***)

*In contrast to this study, prior research conducted in Ghana has yielded dissimilar results, with the most frequently consumed ED brands being identified as Blue Jeans, Lucozade, Rox, Monster, and Red Bull [20,21].*

(***See discussion/knowledge section, pg. 16, line nos. 349-361***)

*EDs are perceived to have several benefits, including increased alertness, improved work or study concentration, energy boost, stress management, and promotion of health and social relationships [15]. This study aligns with previous research [22,27,36,37], indicating a prevailing belief in the benefits of EDs, with the highest perceived advantages being increased work or study concentration (20%), energy boost (81%), and stress management (62.3%). Also, this study reported perceived adverse effects including insomnia (60.60%), and restlessness (51.40%) as commonly reported adverse effects of EDs, similar to work by Casuccio et al. [26]. While perception is known to be autonomous concerning thought, knowledge may lead to an intentional intervention in the process of achieving a percept [38]. This suggests perceptions may be influenced by external factors, notably well-targeted advertisements and recommendations from friends and family, which contribute to shaping a positive view of EDs among consumers. Understanding these external influences is crucial for devising effective health communication strategies and interventions to promote informed and balanced perspectives on ED consumption.*

(***See discussion/knowledge section, pg. 16, line nos. 362-370***)

*Further, upon analysing the data, it is evident that a large proportion of the sample population in this study has poor knowledge of EDs, accounting for 83.4%. This trend is consistent with another study that also reported a high rate of poor knowledge among participants [19]. However, in contrast to this study Saku et al., [19] did not find a significant relationship between knowledge and ED use. The findings of this study suggest that low knowledge can result in a high prevalence of ED use. Therefore, it is crucial to address issues of misconceptions and lack of knowledge. For instance, information about the adverse effects of EDs can be incorporated into default marketing strategies. Also, the lack of warning labels and inadequate inclusion of risks and side effects in advertisements and messaging contribute to this poor knowledge [19,32,39].*

(***See discussion/attitude and practice section, pg. 16&17, line nos. 377-389***)

*Moreover, an even higher percentage of individuals have ever tried EDs, with nearly the entire study population (98.7%) reporting consumption at some point. This trend is consistent with studies conducted among adolescents in Shanghai, China (70.5%) and in Ho (85.6%) [19,41]. The high prevalence of ever-consuming EDs is attributed to factors such as curiosity and perceived utility [24,36]. The increasing popularity of EDs is also attributed to various factors, including individual factors such as perceived benefits, targeted marketing strategies, and limited knowledge about these beverages [21,32,42]. Additionally, interpersonal factors like peer pressure, social image, and parental influence also play a role, along with environmental factors such as affordability, availability, and the lack of policies and regulations [41]. In the context of the HBM [30], these external factors serve as triggers of ED consumption. They can significantly impact individuals’ decision to consume EDs. Therefore, such channels of influence must be monitored to ensure that the general public is well-informed to make healthy choices.*

(***See discussion/attitude and practice section, pg. 17, lines nos. 394-402***)

*The main reasons for consuming EDs reported by the respondents were performance enhancement, stress management, energy boost, and increased work or study concentration. In similar studies conducted among drivers, students and athletes, it was reported that EDs were used to manage fatigue, stay awake, and concentrate for extended periods [19,49–51]. Many ED users appreciate their ability to reduce sleep hours, enhance concentration, boost energy, and serve as refreshments. Particularly, workers use EDs to enhance performance in the workplace, while students use them to cope with academic pressure [19,50,51]. Withdrawal symptoms were reported by ED consumers once the effects wore off. This potentially leads to dependence and workplace accidents that is a risk to themselves and others [52,53].*

(***See discussion/attitude and practice section, pg. 17&18, lines nos. 416-422***)

*EDs are particularly popular among young individuals, especially those in their early twenties [32,36,54]. This can be attributed to the energy-demanding nature of that age group [55]. Young people's curiosity [56] and inclination to follow trends also contribute to their higher consumption of EDs compared to older individuals [43]. Additionally, males generally consume EDs more frequently, particularly at night, and experience more side effects. This aligns with the marketing strategies of EDs, which often target young males and emphasize activities associated with youth and masculinity, such as sports. [6,31,36,54,57,58].*

(***See discussion/attitude and practice section, pg. 18, line nos. 424&425***)

*Education level is associated with ED use [26,59]. Accordingly, ED consumption is higher among those with low education levels, and people who work or study long hours [19].*

***(See discussion/attitude and practice, pg. 18, line nos. 438-449)***

*According to this study, smokers make up a small proportion of ED consumers. The lack of association between alcohol consumption and ED use is attributed to taste preferences and concerns about the impact of combining the two for performance [62] - one of the primary reasons for consuming EDs among the participants in this study. Similar to previous studies by Casuccio et al. [26]. and Chang et al. [15], no significant associations were found between ED use and these variables. Furthermore, this study revealed that easy access to EDs corresponds to higher consumption rates. EDs are readily available and easily accessible [63] to children and young adults, with numerous brands found in every shop that sells beverages [47,64]. Additionally, there is a lack of government regulations governing the sale and marketing of EDs. This unrestricted promotion and utilization of EDs contribute to their increasing appeal, popularity, and availability [5]. The knowledge or experience of side effects also plays a role in influencing the use of EDs [64].*

(***See discussion/predictors of ED consumption, pg. 19, line nos. 497-499***)

*However, generally, ED consumption decreases with increasing age [40,79,80]. Marital status is a predictor of ED consumption as well [81].*

7. **Make the work aligned to the health belief model**

The discussion has been aligned more explicitly with the Health Belief Model (HFM), providing a framework for understanding energy drink consumption in the context of health beliefs and perceptions.

(***See discussion/knowledge, pg. 15, line nos. 318-321***)

*Within the framework of the Health Belief Model (HBM) [30], this underscores the pivotal role of social influences, acknowledging their impact on interpersonal relationships and, consequently, health-related decision-making processes.*

(***See discussion/attitude and practice, pg. 17, line nos. 386&387***)

*In the context of the HBM [30], these external factors serve as triggers of ED consumption.*

(***See discussion/attitude and practice, pg. 17, line nos. 403&404***)

*This aligns with the HBM [30] consideration of perceived benefits for users and an understanding of perceived susceptibility and severity for non-users.*

(***See discussion/attitude and practice, pg. 17, line nos. 411 & 412***)

*Compared to the perceived benefits highlighted by the HBM [30], the adverse effects and the influence of personal experiences, also clarify potential barriers to ED consumption.*

(***See discussion/attitude and practice, pg. 18, line nos. 435&436***)

*This aligns with the HBM [30], where the acknowledgement of modifying variables is pivotal in comprehending their role in shaping health-related behaviors.*

8. **The paragraph before socio-demographic must be sent to the Methodology portion.**

The paragraph before socio-demographic details in the results section has been moved to the methodology section for better organization and flow *(****See methodology section, pg. 6, line nos. 167-169****)*.

*The data gathered explored respondents' socio-demographic characteristics, knowledge, attitudes, and practices regarding EDs. Analysis was done using various statistical tests, including T-tests, chi-square tests, Pearson's r correlation, and logistic regression, with a significance level of 0.05.*

**REVIEWER 2**

**Thank you for the opportunity to review your work. The article provides valuable insights into the consumption patterns and perceptions of energy drinks among the youth in the Tamale Metropolis. I find the work valuable and pertinent to the research landscape around energy drinks however, will benefit from some minor revisions. Find below my general and specific comments to improve the quality of your work.**

We sincerely appreciate your thorough review and valuable feedback on our manuscript. Your very specific insights have significantly contributed to refining the quality and clarity of our work. Below are the revisions made in response to your comments:

General Comments:

a. **Ensure consistency in terms used throughout the paper. For example, "energy drinks" and "EDs" are used interchangeably. While abbreviations are acceptable, introduce them properly the first time and use them consistently thereafter.**

We have ensured consistency in terms, abbreviations, and formatting throughout the paper.

b. **Ensure that all claims, especially those that are not common knowledge, are backed by appropriate references.**

All claims, especially statistical ones, are now properly cited.

c. **Maintain consistency in terms, abbreviations, and formatting throughout the paper.**

We have ensured consistency in terms, abbreviations, and formatting throughout the paper.

d. **Some sentences are lengthy and could be split for better readability and understanding.**

Sentences have been split for better readability and understanding.

e. **The methods section, in particular, could benefit from more detailed explanations to demonstrate the rigor of the research process.**

The methods section has been revised to provide more detailed explanations demonstrating the rigor of the research process.

f. **Ensure that all claims, especially statistical ones, are properly cited. For instance, statements like "62% of student-athletes consume at least one can of energy drink per week" should have a reference.**

We have included a references for statements like "62% of student-athletes consume at least one can of energy drink per week" (***See introduction section, pg. 3, line nos. 78&79***)

*A survey revealed that 62% of student-athletes consume at least one can of ED per week [20].*

# **Specific Comments:**

1. **The sentence "Energy drinks were consumed by the majority of the youth" is redundant given the statistics provided earlier in the abstract. Consider removing or rephrasing.**

The redundant sentence in the abstract has been deleted (***See abstract section, pg. 2***).

*~~"Energy drinks were consumed by the majority of the youth"~~*

2. **The statement "Ingredients commonly found in energy drinks include caffeine, guarana, ginseng, taurine, glucose, L-carnitine, glucuronolactone, and B vitamins" could benefit from a citation.**

The statement about energy drink ingredients now includes a citation ***(See introduction section, pg. 3, line nos. 57&58)***.

*Ingredients commonly found in EDs include caffeine, guarana, ginseng, taurine, glucose, L-carnitine, glucuronolactone, and B vitamins [4].*

3. **The transition between the global context of energy drinks and the specific situation in Ghana could be smoother. Consider adding a sentence to bridge the two sections.**

The transition between the global context and the specific situation in Ghana has been smoothed in one sentence ***(See introduction section, pg. 3, line nos. 77&878)***.

*In the specific context of Ghana, a nation within a sub-region grappling with an uptrend in diet-related diseases [18], there has been a discernible uptick in ED consumption [19].*

4. **"Throughout history, humans have sought physical and cognitive enhancements to tackle challenging tasks." This is a broad statement. Consider adding a citation or example for context.**

The broad statement regarding the history of seeking physical and cognitive enhancements now includes a citation for context ***(See introduction section, pg. 3, line nos. 54&55)***.

*Throughout history, humans have sought physical and cognitive enhancements to tackle challenging tasks [1].*

5. **"The rise of energy drinks (EDs) and their marketing strategies, aimed at boosting physical and cognitive performance, has led to a significant increase in consumption among young people."**

We have rewritten the sentence in focus by splitting it and including citations for readability, clarity and context ***(See introduction section, pg. 3, line nos. 55-56)***.

*Conveniently, Energy Drink (ED) marketing strategies are centered around physical boosts and improved cognitive performance [2]. Thus, there has been a significant increase in consumption among young people [3].*

6. **"However, marketing campaigns geared towards masculinity have resulted in fewer girls consuming energy drinks than boys [3,4]." This is an interesting point. Consider expanding on how these marketing campaigns are geared towards masculinity for clarity.**

The point about marketing campaigns geared towards masculinity has been expanded for clarity ***(See introduction section, pg. 3, line nos. 60-64)***.

*However, marketing campaigns geared towards masculinity have resulted in fewer girls consuming EDs than boys [5,6]. Over the course of history, sports have served as a domain where masculinity is learned, demonstrated, and perpetuated [7]. Indeed, since their inception in 1987, the marketing of these products has heavily depended on visuals linked with extreme sports and risk-taking behaviours [8,9].*

7. **"The study design was a descriptive cross-sectional." Add more details. Why was this design chosen? What advantages does it offer for this research?**

More details have been added to explain why the descriptive cross-sectional design was chosen, highlighting its advantages for this research ***(See methods section, pg. 4, line nos. 96-105)***.

*The study design was a descriptive cross-sectional. This approach was primarily selected for its suitability in capturing a snapshot of the population's characteristics and behaviours at a specific point in time. This design was deemed appropriate for the research objectives. It allowed for a comprehensive examination of ED consumption patterns among the study participants within the defined timeframe. The reasons for utilizing a descriptive cross-sectional design lie in its efficiency in providing a quick and cost-effective overview of the prevalence and distribution of the studied variables. This approach facilitates the identification of trends, making it particularly valuable for investigating the current landscape of ED consumption in the target population. Additionally, the design enables the simultaneous assessment of multiple factors, contributing to a holistic understanding of ED consumption within the constraints of a singular data collection period.*

8. **"The Tamale Metropolis is an urban area in the Northern Region with a population of about 360,579, 80.8% of which lives in urban areas and a size of 289.58 square miles [15]." This sentence is a bit cluttered. Consider splitting it: "The Tamale Metropolis, located in the Northern Region, has a population of about 360,579. Of this, 80.8% reside in urban areas. The metropolis spans 289.58 square miles [15]."**

The cluttered sentence about the Tamale Metropolis has been split for better readability ***(See methods section, pg. 4, line nos. 107&111)***.

*With a population of about 360,579, the Tamale Metropolis is located in the Northern Region. Of this, 80.8% reside in urban areas. The metropolis spans 289.58 square miles [25].*

9. **"The focus of this research was on individuals aged 15 to 45 years old." Why was this age range chosen? Provide a rationale.**

Specific information about the demographic and perception sections of the questionnaire has been added ***(See methods section, pgs. 4&5, line nos. 116-126)***.

*The focus of this research was on individuals aged 15 to 45 years old. The demographic aged 15 to 45 comprises approximately 63.3% of the population engaged in economic activities which demonstrates financial capability for purchasing EDs [25]. This group, primarily consisting of teenagers and young adults, holds specific beliefs about the perceived advantages of these beverages, influencing their decision to consume them. Given that individuals in this age range are actively exposed to marketing and advertising for EDs and are still in the process of developing their health habits, understanding their consumption patterns becomes crucial. The engagement of adolescents and young adults in sports, extracurricular activities, and social events further underscores the relevance of comprehending the factors influencing their ED consumption. This knowledge is pivotal for promoting healthy behaviors and mitigating potential health risks associated with their perceptions of ED benefits.*

10. **"The study collected data on individual respondents in the Tamale metropolis through semi-structured interviews using a pretested-structured questionnaire administered via face-to-face interviews with the aid of a Computer Assisted Personal Interview (CAPI) device." This is a long sentence. Consider breaking it down for clarity.**

The long sentence about data collection has been broken down for clarity ***(See methods section, pg. 5, line nos. 143-145)***.

*The study collected data on individual respondents through semi-structured interviews. The questionnaire was pretested and then administered via face-to-face interviews with the aid of a Computer Assisted Personal Interview (CAPI) device.*

11. **"The questionnaire covered demographic information, anthropometry, and perception, with the latter section probing respondents' knowledge of energy drinks." What specific demographic information was collected? Were there any specific scales or metrics used for the perception section.**

Specific information about the demographic information collected and scales used for the perception section has been added ***(See methods section, pg. 5, line nos. 146-157)***.

*In terms of demographic information, the study gathered data on respondents' age, gender, marital status, ethnicity, religion, employment status, work intensity, educational level, and smoking and drinking behaviours. Anthropometric measurements included parameters such as height, and weight. The perception section of the questionnaire probed respondents' knowledge of EDs, exploring factors such as awareness, attitudes, and beliefs. The perception section sought to probe respondents' understanding of EDs, including their ingredients, brands, benefits, and adverse effects. Based on the responses, a score was generated, and respondents' knowledge of EDs was eventually categorized as "poor," "good," or "excellent." The response scales and metrics used in the perception section were Likert, multi-choice, and frequency scales. The study employed a combination of closed-ended questions with predefined response options, as well as open-ended questions to capture a comprehensive understanding of respondents' perceptions regarding EDs.*

12. **"Consent was obtained orally. This mode was used to maximize time." Oral consent can be controversial in some research contexts. Elaborate on why this was deemed sufficient and how it was documented.**

We have elaborated on why we deemed oral consent as sufficient and how it was documented below ***(See methods section, pg. 6, line nos. 172-184)***.

*Consent was obtained orally. The decision to obtain oral consent in this research on ED consumption was meticulously considered, factoring in ethical principles and practical considerations specific to the study. Participants were easily accessible in the central business district of the metropolis, facilitating efficient oral consent acquisition during brief interactions. The research, focusing on surveying participants about their ED consumption habits, presented minimal risk to healthy individuals. Given the impracticality of obtaining written consent from a sizable participant pool, oral consent emerged as a more practical alternative, streamlining the consent process. Before data collection, informed consent was obtained from all participants, emphasizing their voluntary participation and the confidentiality of their information. Participants were provided with clear explanations regarding the purpose, procedures, and potential risks and benefits associated with the study. They were also assured of their right to withdraw from the study at any point without repercussions.*

13. **"The results of the study indicated that a large percentage of the respondents, 98.7%, had consumed energy drinks before, while 78.7% currently consume them." Consider adding a comparison to other studies or expected outcomes.**

A comparison to other studies has been added to the sentence about the percentage of respondents consuming energy drinks ***(See discussion section, pgs. 16&17, line nos. 372-381)***.

*In Denmark, consumption prevalence among young adults is low (15.8%) due to these products being relatively new and more expensive, compared to other soft drinks [33]. Contrary to the relatively low consumption rate of EDs in some environments [33,40], this study reveals that a significant proportion (78.7%) of the youth in Tamale currently consume these beverages. This finding aligns with studies conducted in Sicily among adults (78%), Ho, Ghana among commercial drivers (75%) and student-athletes in Ghana (62%) [19,20,26]. Moreover, an even higher percentage of individuals have ever tried EDs, with nearly the entire study population (98.7%) reporting consumption at some point. This trend is consistent with studies conducted among adolescents in Shanghai, China (70.5%) and in Ho (85.6%) [19,41]. The high prevalence of ever-consuming EDs is attributed to factors such as curiosity and perceived utility [24,36].*

14. **"Respondents believed that energy drinks provided additional energy (81.00%) and reduced stress (62.30%). However, they also perceived side effects such as insomnia (60.60%) and restlessness (51.40%)." Consider adding a brief discussion on why these perceptions might exist in your discussion.**

A brief discussion on the perceptions of energy drinks has been included in response to your comment ***(See discussion section, pg. 16, line nos. 349-361)***.

*EDs are perceived to have several benefits, including increased alertness, improved work or study concentration, energy boost, stress management, and promotion of health and social relationships [15]. This study aligns with previous research [22,27,36,37], indicating a prevailing belief in the benefits of EDs, with the highest perceived advantages being increased work or study concentration (20%), energy boost (81%), and stress management (62.3%). Also, this study reported perceived adverse effects including insomnia (60.60%), and restlessness (51.40%) as commonly reported adverse effects of EDs, similar to work by Casuccio et al. [26]. While perception is known to be autonomous concerning thought, knowledge may lead to an intentional intervention in the process of achieving a percept [38]. This suggests perceptions may be influenced by external factors, notably well-targeted advertisements and recommendations from friends and family, which contribute to shaping a positive view of EDs among consumers. Understanding these external influences is crucial for devising effective health communication strategies and interventions to promote informed and balanced perspectives on ED consumption.*

15. **"Although the majority of respondents (83.4%) were unaware of the classification of energy drinks and their ingredients, side effects, and benefits." This sentence seems incomplete. Consider completing the thought, e.g., "...the majority were still consuming them regularly."**

We have rewritten the incomplete sentence about the majority of respondents being unaware of the classification of energy drinks for clarity and readability ***(See abstract section, pg. 2, line nos. 41&42)***.

*Also, the highest proportion of respondents (83.4%) had poor knowledge of EDs. They were unaware of the classification of EDs and their ingredients, side effects, and benefits.*

16. **"Age, marital status, level of education, work intensity, EDs served at gatherings, and knowledge of EDs was significantly associated with ED consumption (p < .05)." Elaborate on each factor's specific impact or correlation strength in your discussion.**

We elaborated on the specific impact or correlation strength of each factor associated with ED consumption in the discussion ***(See discussion section, pgs. 19&20, line nos. 490-530)***.

*The findings of this study show certain factors may influence ED consumption. Consistent with the findings of other studies [76–78], age is a significant predictor of ED consumption. For those in the younger age groups, consumption can be associated with developmental influences on consumption behaviors such as curiosity, addiction, peer influence, high perception of their body’s durability, or low health consciousness. In the older age group, consumption indicates shifts in preferences or lifestyle factors as individuals enter adulthood, greater purchasing power, increased dependence on external sources of energy, and increased energy demands. However, generally, ED consumption decreases with increasing age [40,79,80]. Marital status is a predictor of ED consumption as well [81]. This appears to be the case because relationships with people play a key role in aspects of lifestyle and decision-making. By extension, it impacts nutrition and health-seeking behavior. Individuals with different marital statuses might have distinct motivations for consuming EDs, influenced by factors such as social norms, stress levels, marital conflicts, the stress of a failed marriage and other marriage dynamics. Therefore, it is not out of place that the findings of this study have revealed marital status to be a factor that influences consumption. The intensity or perceived intensity of work done by an individual inadvertently causes the need for more energy to complete tasks, especially for those in the blue-collar job bracket. This is in line with this study’s findings that indicate that people who perceive their work to be low in intensity are less likely to consume EDs. Meanwhile, another study conducted among adolescents contradicts this finding by reporting that ED consumption is highest among those who are less physically active [40]. The difference may be a result of a difference in culture in the way adolescents are raised as well as the span of age categories covered by this study in its study population. Education plays a key role in exposure and understanding of information including the types related to health. And this is demonstrated in the findings - the level of education has a relationship with ED consumption. Those with tertiary education are less likely to consume EDs when compared to those who have never had a formal education [82]. This could mean that the exposure to and ability to seek and understand health information such as what EDs are and what their potential side effects may be, people with formal education are better informed to make the right choices. Those who have not had a formal education are usually unable to verify information, struggle to accept new information, are easily misinformed, accepting of myths, and discard facts in some cases. A related factor of interest is the level of knowledge people have of EDs in general. An understanding of the drinks referred to as EDs, their potential benefits, side effects, and even constituent ingredients could influence the decision to consume ED products. Meanwhile, a great proportion of individuals have low knowledge of EDs [82] Special attention should be given to those with low education status and low health literacy because formal and health education can play a critical role in the way in which people consume EDs. Evidence from other studies shows that higher education is associated with reduced consumption of EDs [58,82]. Consumption of EDs was found to be 84% higher when consumed in private compared to consumption at public gatherings. This may be the case because EDs are served with other drink options. They may be served per person according to budgeting constraints which will impact consumption. Also, on the other hand, it is the private intrinsic needs of individuals that may push them to consume more EDs.*

17. **"Energy drinks have not only been associated with negative health effects and death but have also been linked to high-risk substance use and social deviance such as vandalism and reckless driving, resulting in accidents [8–11]." This is a strong claim. Ensure that the cited references robustly support this statement.**

We have ensured that the cited references robustly support the strong claim about energy drinks being associated with negative health effects and death ***(See introduction section, pg. 3, line nos. 72-74)***.

*EDs have not only been associated with negative health effects and death but have also been linked to high-risk substance use and social deviance such as vandalism and reckless driving, resulting in accidents [4,13–15].*

18. **"Sub-Saharan Africa is experiencing an upward trend in diet-related diseases, including hypertension, obesity, stroke, and heart disease, contributing to the rapid increase in the epidemic of non-communicable diseases." How does this relate to the study's findings? Consider drawing a clearer connection between the broader context and the specific results of this study.**

We deleted the sentence and incorporated it into the next sentence. We hope this better draws a clearer connection between the broader context of diet-related diseases in Sub-Saharan Africa and the study's findings. The resulting thought is quoted below ***(See introduction section, pg. 3, line nos. 77&78)***.

*In the specific context of Ghana, a nation within a sub-region grappling with an uptrend in diet-related diseases [17], there has been a discernible uptick in ED consumption [18].*

19. **"In Ghana, where energy drink consumption is on the rise, a survey revealed that 62% of student-athletes consume at least one can of energy drink per week." What are the implications of this finding, especially in the context of the study's results?**

We discussed the implications of the finding that 62% of student-athletes consume at least one can of energy drink per week ***(See discussion section, pg. 17, line nos. 389-393)***.

*ED consumption among Ghanaian youth is concerning due to its potential health risks [43]. These drinks have high levels of caffeine and sugar, which can cause cardiovascular and neurological issues and contribute to weight gain, obesity, and dental problems [16,44–48]. These findings emphasize the need to address the health implications of ED consumption, particularly for the youth.*

20. **"The overall aim of this study was to evaluate the youth's perceptions, consumption habits, and factors linked with energy drink consumption in the Tamale Metropolis."
    Reiterate the main findings at the beginning of the discussion in relation to this aim to provide a clear summary of the study's contributions.**

We reiterated the main findings at the beginning of the discussion in relation to the aim of the study ***(See discussion section, pg. 15, line nos. 304&305)***.

*Hence, this study aims to evaluate the youth's perceptions, consumption habits, and factors linked with ED consumption in the Tamale Metropolis.*

21. **"This study will make clear enable the understanding of the consumption habits of energy drinks among youth in the Tamale Metropolis will contribute to raising awareness, prompt more research in the area and influence policies and public health actions."
    This sentence is convoluted. Rephrase for clarity: "This study enhances our understanding of energy drink consumption habits among the youth in the Tamale Metropolis. The findings can raise awareness, prompt further research, and influence policy and public health initiatives."**

We rephrased the sentence about the study's contributions for clarity ***(See introduction section, pg. 4, line nos. 91-93)***.

*This study enhances our understanding of ED consumption habits among the youth in the Tamale Metropolis. The findings can raise awareness, prompt further research in this domain, and influence policy and public health initiatives.*

However, marketing campaigns geared towards masculinity have resulted in fewer girls consuming EDs than boys [5,6]. Over the course of history, sports have served as a domain where masculinity is learned, demonstrated, and perpetuated [7]. Indeed, since their inception in 1987, the marketing of these products has heavily depended on visuals linked with extreme sports and risk-taking behaviours [8,9].
